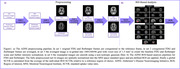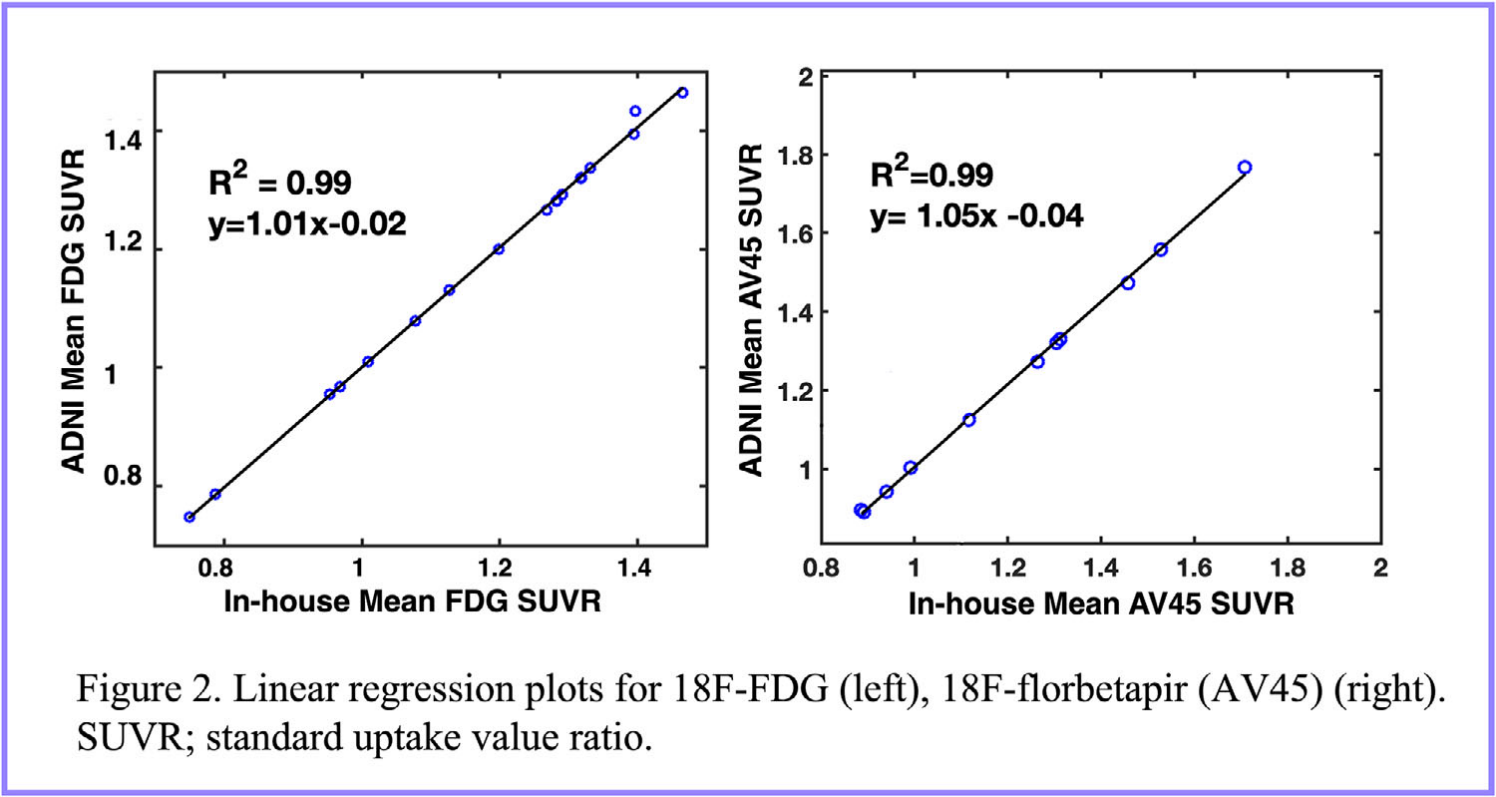# Reproducibility Challenges in Brain PET Data Analysis

**DOI:** 10.1002/alz.093819

**Published:** 2025-01-09

**Authors:** Maryam Naseri, Owen T. Carmichael, Sreekrishna R. Pillai

**Affiliations:** ^1^ Pennington Biomedical Research Center, Baton Rouge, LA USA

## Abstract

**Background:**

While a great deal of recent effort has focused on addressing a perceived reproducibility crisis within brain structural magnetic resonance imaging (MRI) and functional MRI research communities, less attention has been paid to reproducibility of brain positron emission tomography (PET) research.

**Methods:**

We examined the current landscape of factors that contribute to reproducible neuroimaging data analysis, comparing brain MRI to brain PET. The factors included scientific standards, analytic plan pre‐registration, data and code sharing, containerized workflows, and standardized processing pipelines. To demonstrate the positive impact that further developing such reproducibility factors would have on brain PET research, we conducted one case study in which the brain PET processing pipeline developed for one flagship study (Alzheimer’s Disease Neuroimaging Initiative, ADNI) for brain 18F‐florbetapir and 18F‐fluorodeoxyglucose (FDG) analysis was reproduced in our laboratory.

**Results:**

Compared to fMRI, PET research encounters substantial challenges in adopting essential reproducibility practices. While 85% of fMRI studies adhere to the standardized reporting practices, PET is in the early stages of establishing reporting practices, lacking a standardized checklist. Pre‐registration is prevalent for fMRI (57.6%) but notably absent in PET, with no pre‐registered PET studies found on platforms such as the Center for Open Science. The number of shared datasets is higher for fMRI compared to PET, with 92 fMRI datasets and only 9 PET datasets on repositories like OpenNeuro. Moreover, there are very few data repositories for newer PET radiotracers. Containerized software availability also favor fMRI, with platforms like (Brain Imaging Data Structure, BIDS) apps hosting 66% dedicated fMRI tools, while no PET‐specific software is available. Standardized processing pipelines follow the same trend, with fMRI boasting optimized workflows for specific protocols, while PET has few dedicated pipelines and lacks validation studies for existing options. Furthermore, while our case study demonstrated excellent correlation results for both the 18F‐FDG and 18F‐florbetapir methods (Figure 2), the replication of the ADNI PET pipeline posed substantial challenges and implementation delays. These obstacles and delays in execution were due to incomplete documentation and ambiguities in application details.

**Conclusion:**

PET neuroimaging lags behind its MRI‐related counterparts in achieving robust reproducibility.